# The Application of an Emerging Technique for Protein–Protein Interaction Interface Mapping: The Combination of Photo-Initiated Cross-Linking Protein Nanoprobes with Mass Spectrometry

**DOI:** 10.3390/ijms15069224

**Published:** 2014-05-26

**Authors:** Renata Ptáčková, Tomáš Ječmen, Petr Novák, Jiří Hudeček, Marie Stiborová, Miroslav Šulc

**Affiliations:** 1 Institute of Microbiology v.v.i., Academy of Sciences of the Czech Republic, Vídeňská 1083, CZ-14220 Prague 4, Czech Republic; E-Mails: renat.s@seznam.cz (P.R.); tomas.jecmen@centrum.cz (J.T.); pnovak@biomed.cas.cz (N.P.); 2 Department of Biochemistry, Faculty of Science, Charles University, Hlavova 2030, CZ-12843 Prague 2, Czech Republic; E-Mails: jiri.hudecek@natur.cuni.cz (H.J.); stiborov@natur.cuni.cz (S.M.)

**Keywords:** 14-3-3ζ homodimer, protein-protein interaction, photo cross-linking, protein nanoprobe, mass spectrometry

## Abstract

Protein–protein interaction was investigated using a protein nanoprobe capable of photo-initiated cross-linking in combination with high-resolution and tandem mass spectrometry. This emerging experimental approach introduces photo-analogs of amino acids within a protein sequence during its recombinant expression, preserves native protein structure and is suitable for mapping the contact between two proteins. The contact surface regions involved in the well-characterized interaction between two molecules of human 14-3-3ζ regulatory protein were used as a model. The employed photo-initiated cross-linking techniques extend the number of residues shown to be within interaction distance in the contact surface of the 14-3-3ζ dimer (Gln8–Met78). The results of this study are in agreement with our previously published data from molecular dynamic calculations based on high-resolution chemical cross-linking data and Hydrogen/Deuterium exchange mass spectrometry. The observed contact is also in accord with the 14-3-3ζ X-ray crystal structure (PDB 3dhr). The results of the present work are relevant to the structural biology of transient interaction in the 14-3-3ζ protein, and demonstrate the ability of the chosen methodology (the combination of photo-initiated cross-linking protein nanoprobes and mass spectrometry analysis) to map the protein-protein interface or regions with a flexible structure.

## 1. Introduction

The use of photo-initiated cross-linking of protein nanoprobes in combination with mass spectrometry (MS) is an experimental technique suitable for the determination of the assembly of protein complexes in their native states at reasonable time-scales using relatively small quantities of protein [1]. This approach is an alternative to chemical cross-linking, but more powerful due to the absence of limitations in reaction specificity and restrictions on reaction conditions that are inherent to chemical cross-linkers [1].

For this experimental approach, the protein of interest was purified after recombinant expression that incorporated the amino acid residues with a photo-labile group [2]. The incubation and photo-activation of the studied protein produced a mixture of covalently cross-linked homodimers. The abundance of cross-linked species with diverse protein orientations statistically depends on the incidence of these transient interactions in the reaction mixture. The next steps were separation of monomers and homodimers (e.g., by sodium dodecyl sulfate polyacrylamide gel electrophoresis (SDS-PAGE)), digestion of cross-linked species, and analysis of the resulting peptide mixture employing MS techniques. The high accuracy of MS data enables the identification of the unique combination of two cross-linked peptide sequences that conform to protease specificity (e.g., trypsin generates peptides with *C*-terminal arginine or lysine). The deduction of which two amino acid residues participate in the covalent coupling of two cross-linked peptides is based on the acquisition of the tandem mass spectra (MS/MS), which provides structural information about the analyzed species. When photo-amino acid analogs are introduced into the protein sequence, the highly reactive carbene formed after UV-light photolysis of photo-labile diazirine reacts non-specifically, it is able to connect any group, and forms cross-links of zero-length. Because the MS/MS analysis allows identification of amino acid cross-linked residues and the reaction radius of the carbene species is approximately within distances under 5 Å (similarly to the zero-length chemical cross-linker 1-ethyl-3-(3-dimethyl(aminopropyl)) carbodiimide hydrochloride (EDC) [3]), sufficient information to deduce distance constraints between identified residues and to map the interface in the studied protein complex could be obtained. Generally, the experimental approach described in this work offers the ability to study not only homodimer formation, but also to generate oligomers, depending on the subunit arrangements in a protein complex. Because of the method’s ability to promote protein–protein interaction, it also allows for the possibility to characterize the formation of heteromers, the particular identities of which will be dependent on the composition of the studied protein-mixture and the protein–protein functionality.

The 14-3-3 proteins represent a broad class of highly conserved regulatory proteins found in all eukaryotic cells and involved in different cellular pathways. Their main overall function depends on self-assembling into dimers *in vivo* and *in vitro* [4]. This dimerization is mediated by electrostatic interactions between the *N*-terminal regions of two 14-3-3 subunits and can be regulated by site-specific phosphorylation at serine 58 *in vivo* and *in vitro* [5]. The three salt bridges: Arg18–Glu89, Glu5–Lys74, and Asp21–Lys85, as well as several buried polar and hydrophobic residues (Leu12, Ala16, Ser58, Val62, Ile65, and Tyr82) have been described to be involved in the homodimer interface by analysis of a wide range of 14-3-3 homodimer crystal structures [6]. Hydrogen/Deuterium (H/D) exchange techniques revealed the interaction of the *N*-terminal sequence (the first 27 residues of αA-helix) from one subunit with residues 45-98 (αC'-helices and αD'-helices) of the other molecule across the dimer interface. Chemical cross-linking experiments using a zero-length carbodiimide EDC extended the number of amino acid residues detected to be interacting within the studied complex by identifying two alternative salt bridges between Glu81 and either Lys9 or the *N*-terminal amino-group. Molecular modeling was employed to verify the accuracy of the observed chemical cross-links and/or to illustrate their potential structural arrangement, using the published 14-3-3 crystal structure as a starting point for the docking calculations [7]. The resulting model illustrated the feasibility of the observed interaction and visualized its native structure in solution. Therefore, this well-defined homodimeric protein–protein interaction was used to validate the emerging technique of combining photo-initiated cross-linking protein nanoprobes with high-resolution MS and MS/MS technique. Moreover, further amino acid residues participating in transient interactions of this 14-3-3ζ isotype homodimerization were identified.

## 2. Results

### 2.1. Preparation and Characterization of 14-3-3ζ Proteins

**Figure 1:**
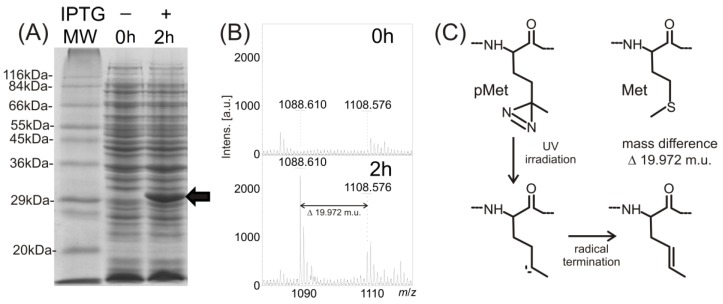
Monitoring of 14-3-3ζ protein expression using 12% sodium dodecyl sulfate polyacrylamide gel electrophoresis (SDS-PAGE) (**A**); incorporation of the photo-labile analog of methionine by matrix-assisted laser desorption ionization-time of flight mass spectrometry (MALDI-TOF MS) (**B**), IPTG, β-d-1-thiogalactopyranoside; MW, molecular weight; a diagram of a reaction mechanism of photolysis (**C**). (**A**) SDS-PAGE of Sigma wide-range molecular weight standards, 14-3-3ζWT expression before induction (0 h) and after induction by β-d-1-thiogalactopyranoside (IPTG) and following incubation (2 h) (black arrow labels the 14-3-3ζWT, Coomassie Brilliant Blue R-250 staining); (**B**) MALDI-TOF MS monitoring of photo-labile methionine analog incorporation within 14-3-3 expressed protein after 0 and 2 h incubation; (**C**) schematic of the photo-methionine (pMet) photolysis reaction mechanism and the structural difference between its termination product and natural methionine (Met).

To carry out the above described experiment, highly purified and well-characterized recombinant proteins, dimeric 14-3-3ζ “wild type” (14-3-3ζWT) and monomeric 14-3-3ζ mutant, prepared with partial incorporation of the photo-labile analog of methionine into the protein sequence, were obtained from *Escherichia coli* (*E. coli*) lysate. In the mutant, the introduction of a negative charge at position 58 mimics Ser58 phosphorylation (14-3-3ζS58D) [8]. They were brought to electrophoretic homogeneity by employing techniques preserving the native structure of these proteins. The incorporation of the photo-labile analog of methionine during recombinant expression in *E. coli* is possible due to adequate structural analogy and the lower specificity of methionine-tRNA synthethase. The matrix-assisted laser desorption ionization-time of flight mass spectrometry (MALDI-TOF MS) analysis of trypsinized proteins using the mass fingerprinting approach confirmed not only the identities of both proteins (see Table 1 for individual values of protein concentrations and sequence coverage), but also verified the successful cleavage of the *N*-terminal histidine tag from protein sequence by thrombin for both protein constructs. The high number of matched peptides resulting from a manual interpretation of all the received *m*/*z* signals indicates the high purity of both analyzed proteins. Because only partial incorporation of the photo-labile methionine analog in place of naturally present methionine within the protein sequence was suggested, the MALDI-TOF analysis of the trypsinized protein nanoprobe was also used to verify sufficient photo-labile analog incorporation. This was confirmed by the detection of signals indicating both tryptic peptides containing methionine and signals corresponding to tryptic peptides with methionine analog (mass shift 19.972 m.u.). For example in preparations of 14-3-3ζWT, the presence of the peptide (159)EMGPTHPIR(167) of the protein sequence should be revealed in the mixture of tryptic peptides by a signal at *m*/*z* 1108.5568. No such signal was detected before protein expression was induced through the addition of isopropyl β-d-1-thiogalactopyranoside (IPTG). After two hours of 14-3-3ζWT protein expression under the IPTG regime, the protein band of relevant molecular weight was detected by SDS-PAGE of the whole cell lysate (see Figure 1A). A signal at *m*/*z* 1108.576 in the MS spectrum of the trypsinized protein band, corresponding to the described tryptic peptide, was also detected (see Figure 1B). Moreover, a signal corresponding to the peptide with the photo-labile methionine analog incorporated into its sequence was detected at *m*/*z* 1088.610 (see Figure 1B). As shown in the reaction mechanism (Figure 1C), the laser, with wavelength 337 nm used for the MALDI ionization process, photolyzed the diazirine in the structure of the methionine analog into reactive carbene that in the high vacuum of the MALDI ionization source, produced the termination product of the bi-radical with a double bond, and the MS signal with mass shift 19.972 m.u., compared to the peptide with natural methionine in its sequence.

**Table 1:** Characterization of purified recombinant human 14-3-3ζ proteins.

Quantity (unit) purified protein	Protein concentration (mg/mL)	Sequence coverage (%)	Matched/searched peptides
14-3-3ζWT	1.45 ± 0.17	74	25/27
14-3-3ζS58D	1.27 ± 0.12	69	27/29

Values of protein concentration in the table are averages ± SD of triplicates; Assay conditions are described in Section 4; Matrix-assisted laser desorption ionization-time of flight mass spectrometry (MALDI-TOF MS) peak lists were searched manually against protein sequences with peptide mass tolerance ± 40 ppm.

### 2.2. Photo Cross-Linking Experiments

**Figure 2:**
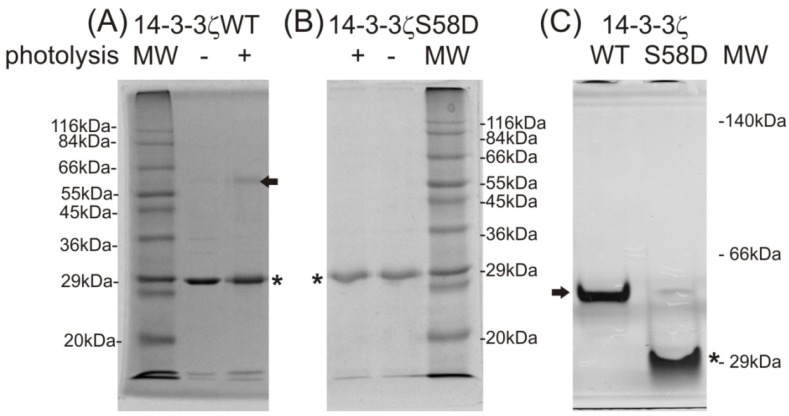
Monitoring of 14-3-3ζ homodimer formation by photo cross-linking using SDS-PAGE (**A**,**B**) and native PAGE (**C**). Non-photolyzed and photolyzed proteins on 12% SDS-PAGE (**A**) 14-3-3ζWT and (**B**) 14-3-3ζS58D; (**C**) non-photolyzed 14-3-3ζWT and 14-3-3ζS58D on 12% basic native PAGE (Coomassie Brilliant Blue R-250 staining, Sigma wide-range molecular weight standards). The black arrow labels the dimeric 14-3-3ζWT and * mark monomeric 14-3-3ζ molecules.

The photo cross-linking reaction of the photo-labile protein nanoprobe with the incorporated photo-labile methionine analog (zero-length photo cross-linker pMet [2]) forms a covalent homodimeric product of 14-3-3ζWT protein (labeled by black arrow in Figure 2A). In agreement with the available literature, the 14-3-3ζS58D mutation significantly reduced homodimer production, introducing one negative charge at this site. This mutation mimics Ser58 phosphorylation by PKA and PKB/AKT1, which regulates a 14-3-3 homodimer assembly in the organism [5,9]. The introduction of three *N*-terminal residues from the cleaved histidine-tag sequence, (−2)GSH(0), to both constructs did not produce any unspecific protein–protein interaction for the 14-3-3ζS58D construct (Figure 2B). Native electrophoresis was employed to demonstrate the native structure and corresponding homodimer formation of 14-3-3ζWT (labeled with a black arrow in Figure 2C) and the monomeric character of 14-3-3ζS58D (labeled by an asterisk in Figure 2C). Therefore, no influence from the incorporation of the photo-labile methionine analog within the sequence was detected, and the native character of both proteins was shown.

### 2.3. The Mass Spectrometry (MS) Analysis of Covalent 14-3-3ζWT Homodimeric Product

The photo cross-linking successfully formed a covalent 14-3-3ζWT homodimeric product detectable by SDS-PAGE. The MALDI-TOF MS analysis verified the identity of the trypsinized 14-3-3ζWT homodimeric product. Moreover, three signals with *m*/*z* values of 1342.744, 1470.824 and 1998.030, specific to the homodimeric product were detected. No such *m*/*z* signal was found in the photolyzed protein nanoprobe band corresponding to both monomers (14-3-3ζWT and 14-3-3ζS58D). Therefore, these three signals correspond to intermolecular cross-links rather than intramolecular ones, and were used to locate the covalently cross-linked regions between two 14-3-3ζ molecules. MALDI-FTICR (Fourier transform ion cyclotron resonance) MS with MALDI ionization and high resolution FTICR detection was used to obtain [M + H]^+^ monoisotopic signals to deduce the unique peptide combination involved in a covalent cross-link.

The values of experimental monoisotopic signals were searched against a database of theoretical monoisotopic masses of cross-linked products: the mass of any 14-3-3ζWT tryptic peptide plus the mass of any 14-3-3ζWT tryptic peptide minus the mass shift of a 19.972 m.u. eliminated during the photo cross-linking reaction. Similarly to MALDI-TOF analysis, the three signals with *m*/*z* values of 1342.7457, 1470.8408 and 1998.0234 were specifically detected in the homodimeric product by MALDI-FTICR analysis. Investigation of these *m*/*z* values revealed the corresponding cross-linked peptides (see Table 2). To increase the resolution power, peptide separation by reverse phase liquid chromatography (LC) was used and on-line coupled to nano-electrospray ionization (ESI) and high resolution FTICR detection (LC-ESI-FTICR MS). In this way, the trypsinized 14-3-3ζWT homodimeric product was analyzed and the covalently cross-linked regions were located. The obtained dataset of experimental *m*/*z* values was automatically reduced to produce output files of monoisotopic masses that were searched against the database of theoretical monoisotopic masses of cross-linked products. Table 2 summarizes all the experimentally determined *m*/*z* values and the corresponding mass error of the calculated and experimental *m*/*z* values (maximally 2 ppm). The sequences of the covalently cross-linked peptides (in the first and the second molecules of the 14-3-3ζWT homodimer) are also shown. The LC-MS analysis confirmed signal values at *m*/*z* 1998.0186 with a lower error of acquired mass. In addition to the described *m*/*z* signals, both techniques (LC-FTICR MS and MALDI-FTICR MS) also revealed several *m*/*z* values corresponding to internally cross-linked peptides or peptides with the termination product of the photolyzed Met analog (e.g., 1881.0186) in the photolyzed 14-3-3ζWT monomer, or 14-3-3ζS58D monomer, respectively. All these signals were removed from 14-3-3ζWT homodimer data sets as intra-molecular cross-links, and were not used for the subsequent analysis of the 14-3-3ζWT homodimer interface. Using the MS/MS analysis, the individual *m*/*z* signals were further resolved to identify the unique covalent cross-links between two amino acid residues involved in the formation of the homodimer.

**Table 2:** Identified intermolecular photo cross-linked products in 14-3-3ζ homodimer between molecule 1 and molecule 2 by * MALDI-FTICR (matrix-assisted laser desorption ionization-time of flight-Fourier transform ion cyclotron resonance) or ^$^ LC-FTICR MS.

[M + H]^+^ experimental	Error (ppm)	14-3-3ζ molecule (1)	14-3-3ζ molecule (2)
1342.7457 *	1.9	4–9 K.NELVQK.A	76–80 K.QQpMAR.E
1470.8408 *	1.8	4–9 K.NELVQK.A	76–80 K.KQQpMAR.E
1998.0186 ^$^/1998.0234 *	0.2 ^$^/2.2 *	(−2)–9 .GSHMDKNELVQK.A	76–80 K.QQpMAR.E

The cross-linked amino acids in the peptide sequence are underlined, pM stands for the reactive photo-labile analog of methionine, and the periods in the sequence delimited the identified peptide.

### 2.4. The MS/MS Analysis of the Covalent 14-3-3ζWT Homodimeric Product

**Figure 3:**
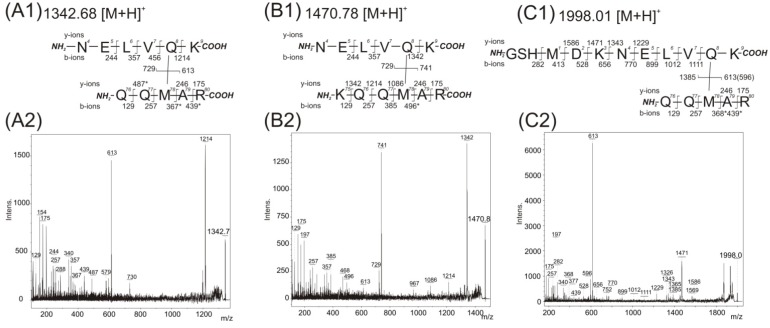
Acquired mass spectrometry (MS)/MS spectra after trypsin digestion of 14-3-3 homodimer band and detected fragment ions corresponding to covalently photo cross-linked structures. The structures of covalently cross-linked Gln8–Met78 (**A1**), (**B1**), and (**C1**) determined from MS/MS daughter ion spectra with detected peaks at *m*/*z* 1342.75 (**A2**), *m*/*z* 1470.84 (**B2**), and *m*/*z* 1998.01 (**C2**), respectively. The residue numbering corresponds with the full-length sequence of the 14-3-3ζ (UniProtKB database accession number P63104). Intens. means absolute intensity; asterisks marks the secondary fragmentation after inter-peptide cross-linked bond cleavage.

The MALDI-TOF/TOF instrument was used to acquire the MS/MS data from the *m*/*z* signals shown in Table 2 after off-line separation of peptides. The interpretation of fragment peaks in the MS/MS spectrum allows for the location of interacting amino acid residues covalently connected by the photo cross-linking reaction. The first and the second row in Table 2 show the contact of the pMet78 residue with a residue within the protein sequence at position 4–9 (peptide NELVQK). The contact of pMet78 residue with the Gln8 residue was confirmed by manual interpretation of recorded MS/MS spectra with its value identified at *m*/*z* 1342.7457 (Figure 3A). The two major fragment ions are present in this spectrum. The first one, *m*/*z* 613 with the complementary ion at *m*/*z* 729, corresponds to the fragmentation of the covalent bond between two cross-linked peptides (76)QQpMAR(80) and (4)NELVQK(9). The second most abundant fragment at *m*/*z* value at 1214 belongs to *b*-ions series (244, 357, 456 and 1214), and demonstrates the covalent modification of the Gln8 residue with the peptide (76)QQpMAR(80) (the signals at 244, 357 and 456 correspond to fragmentation of unmodified amino acids in the sequence (4)NELV(7)).

The MS/MS spectrum (Figure 3B with incorporated fragmentation scheme) of the observed *m*/*z* value 1470.8408 shows the same group of interacting amino acids, pMet78 and Gln8. The observed *m*/*z* difference is explained by the presence of the peptide sequence (75)KQQpMAR(80) formed by trypsin protease miscleavage at the *C*-terminus of Lys75 residue. Also, two other major fragments are detected in this spectrum. The first one, *m*/*z* 741 with a complementary ion of *m*/*z* 729, corresponds to fragmentation of the covalent bond between the two cross-linked peptides (75)KQQpMAR(80) and (4)NELVQK(9). The second value of *m*/*z* 1342 belongs to *b*-ions series (244, 357, 456 and 1342), and again demonstrates the covalent modification of the Gln8 residue with the (75)KQQpMAR(80) peptide (the signals at 244, 357 and 456 correspond to fragmentation of unmodified amino acids in the sequence (4)NELV(7)). The MS/MS spectrum of the identified *m*/*z* value at 1470.8408 contains the full *y*-ion series of (75)KQQpMAR(80) (*m*/*z* 1342, 1214, 1086, 246, 175). This indicates the modification of the pMet78 residue by peptide (4)NELVQK(9) (Figure 3B with incorporated fragmentation scheme).

The *m*/*z* signal at 1998.0186 in the third row of Table 2 can be interpreted as either of two possible cross-links, because each peptide contains a methionine amino acid. The first probably suggests the previously described pMet78 and Gln8 contact. In the second the pMet1 residue can bind to any residue presented within sequence 76–80. The first interpretation, pMet78 and Gln8 contact, is supported by the MS/MS spectrum acquired from a sample that was photolysed with in the absence of glycerol (Figure 3C). The most abundant fragment ion at *m*/*z* 613 with complementary ion at *m*/*z* 1385 corresponds to fragmentation of the covalent bond between two cross-linked peptides (76)QQpMAR(80) and (−2)GSHMDKNELVQK(9). The identified fragments (1111, 1012, 899, 770, 656, 528, 413) confirmed unmodified Met1 (covered sequence (−2)GSHMDKNELV(10)). Moreover, a covalent bond between pMet78 and Gln8 with no modification of Met1 is supported by the presence of fragments 1586, 1471, 1343 and 1229. A similar MS/MS spectrum with a corresponding interpretation of amino acid contact was also obtained with the presence of 150 mM NaCl during photolysis.

**Figure 4:**
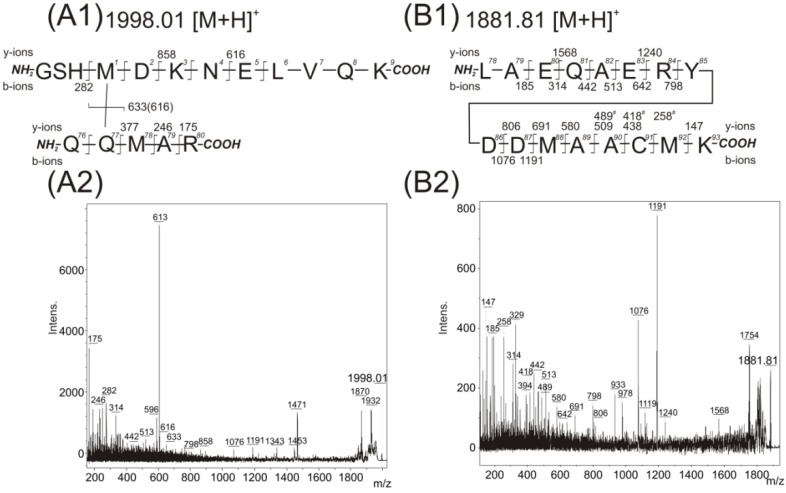
Acquired MS/MS spectra of detected peaks at *m*/*z* 1998.01 (**A2**) and *m*/*z* 1881.87 (**B2**); and structural interpretation of potentially covalently cross-linked Met1–Gln77 (**A1**) and peptide Leu15–Lys30 (**B1**). The residue numbering corresponds with the full-length sequence of the 14-3-3ζ (UniProtKB database accession number P63104 [10]). Intens. means absolute intensity.

Additional tests were done, separately, in the presence of two compounds (10% glycerol (*v*/*v*) and 150 mM NaCl) during photolysis, because both are usually used in protein samples to protect them during storage at −80 °C. No influence of 150 mM NaCl was observed on the MS/MS spectra of all three *m*/*z* signals (1342.74, 1470.84 and 1998.01). The presence of 10% glycerol (*v*/*v*) had no effect on MS/MS spectra at *m*/*z* signal 1342.74 and 1470.84, but surprisingly, was found to have an effect on the MS/MS spectra at *m*/*z* signal 1998.01, revealing additional peaks (Figure 4A). The highly abundant fragments in this MS/MS spectrum corroborated the contact between pMet78 and the Gln8 (similarly to Figure 3C). But some of the observed low intensity fragments could also be interpreted as the existence of a covalent bond between pMet1 and Gln77. The fragment with *m*/*z* value 633 could suggest the presence of the peptide (76)QQMAR(80) with unmodified methionine, the fragments of the *y*-ions series (*m*/*z* values 282, 858 and 616) could indicate the covalent modification of pMet1 with the peptide (76)QQMAR(80). Similarly, the *y*-fragments, corresponding to peptides sequenced from *C*-termini, up to the value of *m*/*z* 377 could demonstrate the absence of modification of the peptide (78)MAR(80). However, several abundant fragments cannot be interpreted according to the proposed structures shown in Figure 3C1 or Figure 4A1. Therefore a detailed reanalysis was made of the LC-FTICR MS data at the retention time corresponding to the elution of the *m*/*z* signal 1998.0186 to determine if any co-eluted peptides could have contaminated the spectra. The partially co-eluted peptide (12)LAEQAERYDD(pM)AAC(pM)K(27) with an *m*/*z* value of 1881.8459 containing two methionine in its sequence (one of which being the photo-analog) and a species with an *m/z* value of 1861.8735 (with two incorporated photo-analogs) were detected (Table 3). The MS/MS of the species with *m*/*z* value 1881.84 was acquired (Figure 4B) and an almost complete *b*-ion series with *m*/*z* values up to Asp21, and with two major fragments with *m*/*z* values of 1076 and 1191 was detected. The latter two *m*/*z* values were surprisingly also detected in the MS/MS spectrum of *m*/*z* signal 1998.01. There is no simple explanation for such an event. Nevertheless, the *m*/*z* values of both peptides (*m*/*z* 1881.8459 and 1861.8735 are so distinct from those of the interpretation of the MS/MS spectrum for *m*/*z* 1998.01 that their mutual contamination can be excluded. Moreover, no fragments with *m*/*z* values 282, 616, 633, 858 and 377 were found in the MS/MS spectrum of *m*/*z* signal 1881.84. Further study revealed a weak signal of a third co-eluted peptide at *m*/*z* 1995.8791 (Table 3 row 3), suggesting the presence of the peptide (12)LAEQAERYDD(pM)AAC(pM)K(27) with a covalently bonded monosodiated glycerol molecule on one of the methionine photo-analogues in the sequence. Because the MS/MS spectrum of *m*/*z* 1998.01 was acquired with a wider *m*/*z* window of 6 m.u. due to the low intensity of this signal in MS mode, the fragments of the proposed peptide with *m*/*z* value 1995.87 should be present in this MS/MS spectrum. The presence of fragments with *m*/*z* values 314, 442, 513, 642, 798, 961, 1076 and 1191 strongly confirm the presence of the sequence (12)LAEQAERYDD(21). Therefore, to correctly interpret the obtained MS/MS spectrum of 1998.01, the chromatographic separation of the species with *m*/*z* value 1995.87 from the species with *m*/*z* value 1998.01 is necessary. Unfortunately, no chromatographic conditions with adequate separation of both signals were found.

**Table 3:** Photo-initiated cross-linked products (potentially intramolecular or modified) identified in 14-3-3ζ monomer (molecule 1) by * MALDI-FTICR or ^$^ LC-FTICR MS.

[M + H]^+^ experimental	Error (ppm)	14-3-3ζ molecule (1)	14-3-3ζ molecule (2)
1861. 8735 ^$^^,^*	1.0 ^$^	12-27+CAM K.LAEQAERYDDpMAACpMK.S	–
1881.8459 ^$^^,^*	0.9 ^$^	12-27+CAM K.LAEQAERYDD(pM)AAC(pM)K.S	–
1995.8791 ^$^^,^*	1.2 ^$^	12-27+CAM+GONa K.LAEQAERYDD(pM)AAC(pM)K.S	–

Cross-linked amino acids in the peptide sequences are underlined; pM denotes the reactive photo-labile analog of methionine; (pM) stands for two alternatively incorporated reactive photo-labile analogs of methionine; GONa marks the covalent modification of a photolysed analog of methionine with a monosodiated molecule of glycerol, identified periods in the sequence delimit the observed peptide; CAM marks the modified cysteine (by carbamidomethyl group).

The *m*/*z* value 1881.8439 (row 2 of Table 3, Figure 4B) allows for the potential presence of an intramolecular cross-link. However, detailed analysis of the fragments in the acquired MS/MS spectrum revealed no such intramolecularly linked pMet residue and the existence of a mixture of two species (the first species with presence of pMet at position 22, the second one at position 26). The fragments with *m*/*z* values of 438, 509 and 580 demonstrated the existence of a double bond on the methionine analog at position 22. On the other hand, the fragments with *m*/*z* values of 258, 418 and 489 showed the presence of a double bond on the methionine analog at position 26. The accessibility of one or both Met residues at positions 22 and 26 to the solvent also demonstrate the identification of covalent modification of this residue by glycerol in *m*/*z* signal 1995.8791 (row 3 of Table 3).

To summarize, in agreement with previously performed molecular dynamic experiments [7], one detected covalent cross-link: Gln8–pMet78 was found within the 14-3-3ζWT homodimer complex interface by MS/MS experiments. At the same time these results verified the applicability of the described methodology (the combination of photo-initiated cross-linking protein nanoprobes and mass spectrometry analysis) to map the protein-protein interface.

## 3. Discussion

### 3.1. Description of Interaction in 14-3-3ζWT Homodimer

**Figure 5:**
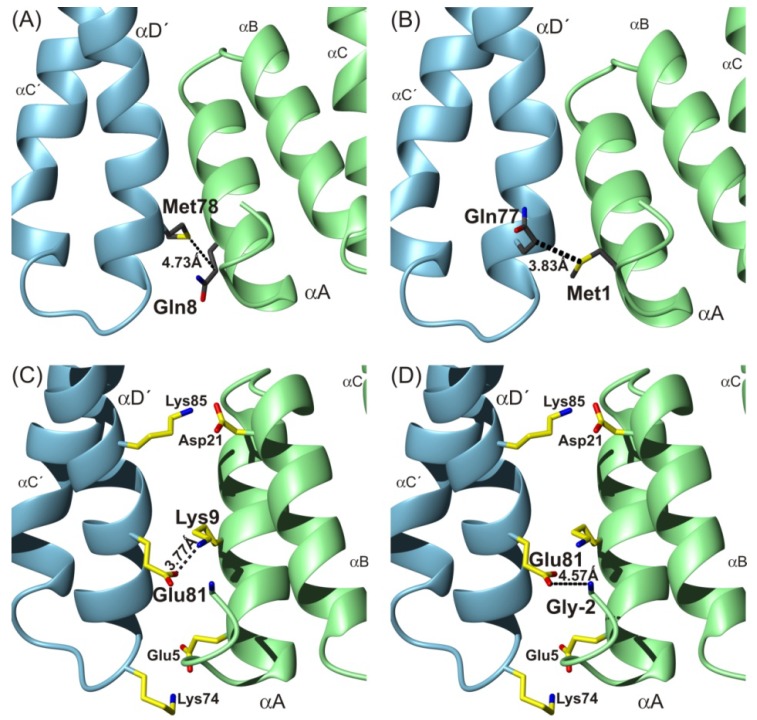
Illustration (top view) of contacts detected by photo and chemical cross-linking techniques. The detected contacts are shown in the adapted 3rdh X-ray [7] structure of 14-3-3ζ homodimer using molecular dynamic technique: Gln8–Met78 (**A**); potential Met1–Gln77 (**B**); Lys9–Glu81 (**C**); and *N*-terminal amino group–Glu81 (**D**). The two salt bridges Glu5–Lys74 and Asp21–Lys85 [6,12] are also pictured in parts (**C**,**D**).

We have identified two amino acids involved in 14-3-3ζWT homodimerization contact (Gln8–Met78) that are situated within two regions previously detected by Hydrogen/Deuterium exchange MS analysis as contact interfaces [7]. These data are also in agreement with previously described findings that the αA'-helix region on the first 14-3-3 molecule interacts with the αD'-helix region on the second molecule [4,11]. Our findings from the photo-initiated cross-linking experiment are also supported by the previously observed salt bridges involving Glu5–Lys74, Arg18–Glu89 and Asp21–Lys85 [6,12] together with Lys9–Glu81 or *N*-terminal amino-group–Glu81 covalent linkages detected by chemical cross-linking using EDC and MS analysis [7]. To explain the potential structural arrangement and to illustrate the observed interactions, we applied the model of the 14-3-3 homodimer [7] derived from molecular calculations starting from the published 3rdh PDB file (Figure 5, It employs molecular dynamic calculations with constraint distances derived from EDC chemical cross-linking data) [13]. The distances obtained from this model for the covalent photo-initiated cross-links described in this paper (Figure 5A) were 4.73 Å for Gln8(Cγ)–Met78(S) and 8.70 Å for Gln8(Cα)–Met78(Cα). The determined values of atom distances cross-validated the structural data sets obtained from chemical and photo-initiated cross-linking experiments (these employing differing mechanisms of cross-link formation), and at the same time cross-evaluated both approaches with each other. Therefore, the agreement of both independently acquired results and determined constraint distances with one resulting structure, and the consensus with Hydrogen/Deuterium exchange mass spectrometry support the natural existence of all observed interactions: Gln8–Met78, Lys9–Glu81, Glu81–*N*-terminus.

### 3.2. The N-Terminal Amino-Group Interaction within 14-3-3ζWT Homodimer Interface and Its Hypothesized Role

The previously obtained molecular dynamic results also allow for the discussed contact between Met1 and Gln77 (Figure 5B) (3.83 Å for Met1(S)–Gln77(Cγ) and 8.39 Å for Met1(Cα)–Gln77(Cα)) and our previous results from chemical cross-linking experiments show the interaction of the *N*-terminal amino-group with Glu81 (Figure 5D) [7] and indicate expected *N*-terminal flexibility. However, the interpretation of the MS/MS spectra of the photo cross-link (*m*/*z* value 1998.01) was not unambiguous and whether such interaction occurs naturally is questionable. The data supporting Met1–Gln77 is weak, because an insufficient number of low intensity fragments confirming the existence of the covalent bond between pMet1 and Gln77 were observed. Moreover, the lack of glycerol in photolyzed protein (sample with 0 or 150 mM NaCl) led to the disappearance of the fragments with *m*/*z* values 282, 616, 633, 858 and 377, which suggests two explanations. The first one proposes the influence of glycerol in solution on *N*-terminal flexibility (the formation of Met1–Gln77 interaction) or, more probably covalent glycerol modification of peptide (12)LAEQAERYDD(pM)AAC(pM)K(27) with exposure of the *N*-terminus to solvent with no intermolecular interaction. Because only a low intensity of fragments was obtained, we are inclined to interpret the potentially found contact between Met1 and Gln77 as statistically low in abundance (higher energy state of a smaller population). We cannot exclude a false positive interpretation of this contact either, due to presence of fragments of co-eluted peptide 12–27 with glycerol modification. Moreover, the both cross-linking experiments (photo and chemical) were performed with 14-3-3 protein constructs (14-3-3ζWT and 14-3-3ζS58D) that contain three *N*-terminal amino acid residues remaining from the histidine tag, (−2)GSH(0). These residues did not reveal any non-specific interaction on native basic PAGE electrophoresis, or in cross-linking and H/D exchange experiments. But we cannot exclude the possibility that the Met1–Gln77 cross-link does not exist in the native protein without (−2)GSH(0). Therefore, to confirm our previous hypothesis that the transient interactions between the positive charge on the 14-3-3 protein *N*-terminus and any negative amino acid residue occurring in the αC'-helix and αD'-helix regions offer a potential contact within 14-3-3ζ homodimer complex under the native conditions (e.g., in solution) more experiments have to be performed. Although the functionality of the proposed *N*-terminal amino-group interaction in the 14-3-3 homodimerization process has been previously discussed [7] (*N*-terminal acetylation is thought to increase 14-3-3 isoform half-life by protecting the *N*-terminus from degradation [14], does not have a known effect on 14-3-3 protein functions [15], and can regulate the protein–protein interactions [16]), the experimental evidence of the potential role of acetylated *N*-terminus has not yet been confirmed.

The prepared photo-initiated cross-link protein nanoprobe should provide a suitable tool to explore the structure-function relationships of this 14-3-3 isoform and potentially confirm or exclude the modulatory role of the positively charged *N*-terminal amino-group for the 14-3-3 protein function in regulatory processes in the organism. The reported methodology offers the presence of a reactive species (pMet) at the *N*-terminal position to confirm its interaction within the 14-3-3 homodimer interface or its accessibility to the solvent (through detection of its modification when a quenching agent is introduced into the solvent). However the presence of glycerol in samples with proteins that contain three *N*-terminal amino acid residues remaining from the histidine tag, (−2)GSH(0) did not reveal any detectable covalent modification of pMet1 as was observed for Met22 and Met26 (signal at *m*/*z* 1995.87). The only effect reported in the literature on physiological regulation of 14-3-3 was the suppression of its homodimerization induced by specific Ser58 phosphorylation [8,17].

## 4. Experimental Section

### 4.1. Materials and Reagents

The expression plasmids for 14-3-3ζ proteins cDNA were kindly gifted by Tomas Obsil of Charles University in Prague, Czech Republic. Amino acids and reagents for Dulbecco’s Modified Eagle Medium (DMEM)-Limited Medium, bovine serum albumin, dithiotreitol (DTT), boric acid, formic acid, isopropyl β-d-1-thiogalactopyranoside (IPTG), methionine, acrylamide, sodium dodecyl sulphate, thrombin and trifluoroacetic acid were purchased from Sigma Chemical Co. (St. Louis, MO, USA). Coomassie Brilliant Blue R-250, EDTA sodium salt, glutathione, iodoacetamide, Tris(2-carboxyethyl) phosphine hydrochloride (TCEP), and TrisCl were from Fluka Chemical Co. (St. Louis, MO, USA). Bicinchoninic acid and photo-methionine analog (pMet) were from Pierce (Rockford, IL, USA), trypsin from Promega (Madison, WI, USA), acetonitrile and water of LiChrosolv quality from Merck (Darmstadt, Germany), OligoTM R3 Bulk Media from Applied Biosystems by Life Technologies (Grand Island, NY, USA) and Ni Sepharose High Performance from GE Healthcare (Pittsburgh, PA, USA). The other chemicals were obtained from Lachema (Brno, Czech Republic).

### 4.2. Expression and Purification of 14-3-3ζ Proteins and Their Characterization

The studied human 14-3-3ζ proteins, wild type (WT, UniProtKB database sequence P63104 [10]) and its single-amino acid mutant Ser58Asp, denoted as S58D, were prepared from *E. coli* BL21 (gold) cell lysate as the histidine-tagged recombinant proteins as described previously [7,18,19]. Briefly, the chelating and ion-exchange chromatography, Ni Sepharose High Performance chelating media (GE Healthcare), was applied according to the standard protocol and the purification process was finalized by thrombin (Sigma) cleavage of the histidine-tag, followed by separation of this peptide from the protein using the Ni Sepharose column. The resulting 14-3-3ζWT and 14-3-3ζS58D proteins contain three *N*-terminal amino acid residues remaining from the histidine tag, (−2)GSH(0). The recombinant protein expression was carried out over 2 h of cultivation (4 × 40 mL DMEM-Limited Medium supplemented with photo-methionine analog (pMet, Pierce) after *E. coli* (BL-21 gold) culture growth in LB-medium till OD_600nm_ values were approximately 0.6 and after rigorous washing by sterile 10 mM phosphate buffer (pH 7.7) with 1 mM EDTA (Fluka).

### 4.3. 14-3-3ζ Protein Characterization and Native Polyacrylamide Gel Electrophoresis (PAGE) Electrophoresis

One-dimensional electrophoresis in the presence of SDS-PAGE was used to prove protein homogeneity and purity [20]. Each protein band was analyzed by mass spectrometry after trypsin (Promega) digestion to confirm the sequences of all proteins [21]. Protein concentrations were assessed using bicinchoninic acid (Pierce) and with bovine serum albumin (Sigma) as a standard [22]. The native state of both 14-3-3ζ protein (transient homodimeric interaction for WT and monomeric behavior for S58D), was demonstrated by native PAGE electrophoresis using 5 µM protein concentration containing 20 mM Tris–HCl buffer (pH 7.5), 1 mM EDTA, 1 mM DTT and 10% glycerol (*v*/*v*). The protein mixture was pre-mixed with electrophoretic sample buffer and separated on 12% PAGE in the 90 mM Tris–borate buffer system and 1 mM EDTA (pH 8.0) using 200 V/15 cm for 2 h at 4 °C. The protein bands were visualized using 0.25% (*w*/*v*) Coomassie Brilliant Blue R-250.

### 4.4. Preparation, Separation and Proteolysis of Cross-Linking Products

The photo cross-linking reaction was carried out in a total volume of 50 µL with 2 µM 14-3-3ζ protein in a solution containing 20 mM Tris–Cl buffer (pH 7.5), 1 mM EDTA, 1 mM DTT at room temperature employing 3 min and 15 s of UV-irradiation in a quartz tube (Oriel photolyser equipped with the Hg-Arc lamp emitting at 254 nm). Alternatively, the addition of 10% glycerol (*v*/*v*) or 150 mM NaCl was used in the photo cross-linking reaction mixture to test their influence on structure assembly of *N*-terminal part. Immediately after the irradiation, reduced glutathione (150 µM) was added to halt the cross-linking reaction and the components of the reaction mixture were separated using SDS-PAGE (a 12% polyacrylamide gel). Protein bands were visualized using 0.25% (*w*/*v*) Coomassie Brilliant Blue R-250. The excised protein spots from the SDS-PAGE gel were de-stained, all cysteine residues were modified using Tris(2-carboxymethyl) phosphine hydrochloride and iodoacetamide (Fluka), and processed for MALDI-TOF mass spectrometry by in-gel digestion with trypsin (Promega) as described previously [21]. The mixture of extracted peptides was applied to a peptide micro-trap microcolumn (Michrom Bioresources, Bruker Daltonics, Bremen, Germany) to remove any salts and buffer components prior to LC-MS analysis.

### 4.5. LC-FTICR (Liquid Chromatography-Fourier Transform Ion Cyclotron Resonance) MS Analysis

The peptides eluted from the micro-trap were dried in a rotation vacuum concentrator (SPEED-VAC, Savant by Thermo Fisher Scientific, Waltham, MA USA) and dissolved in 5% (*v*/*v*) aqueous acetonitrile (Merck) containing 0.1% (*v*/*v*) formic acid (Merck) under 15 min sonication. One portion (5 µL) was loaded onto a reverse-phase column (Magic C18, 0.3 mm diameter, 150 mm long, 5 µm particle size, 200 Å pore size, Michrom Bioresources) and eluted using the mobile phase gradient: solvent A, 0.1% (*v*/*v*) formic acid in 5% (*v*/*v*) aqueous acetonitrile; solvent B, 0.08% (*v*/*v*) formic acid in 90% (*v*/*v*) aqueous acetonitrile; gradient (in % of buffer B), 0% for 1 min, 0%–15% (*v*/*v*) over 4 min, 15%–60% (*v*/*v*) over 30 min, 60%–100% (*v*/*v*) over 5 min; flow rate, 3 µL/min. Chromatographic separation was performed at 20 °C using an Ultimate 1000 capillary HPLC system (LC Packings, Nieuwerkerk a/d IJssel, The Netherlands). The column effluent was directly loaded into the Solarix 12T FTICR mass spectrometer equipped with a 12 T superconducting magnet (Bruker Daltonics, Bremen, Germany). Positive ion MS spectra were acquired in a broad *m*/*z* range (300–2000) [23]. Each received monoisotopic mass was automatically matched to the theoretical library of cross-linked, modified and/or unmodified proteolytic products using protein sequences from the UniProtKB database (P63104) with the additional three *N*-terminal residues (−2)GSH(0).

### 4.6. MALDI (Matrix-Assisted Laser Desorption Ionization)-FTICR MS and MALDI-TOF (Time of Flight)/TOF MS/MS Analysis

One portion (4 µL) of the extracted peptides from in-gel trypsin digestion was diluted by aqueous 0.1% (*v*/*v*) trifluoroacetic acid to the desired final concentration 5% (*v*/*v*) of aqueous acetonitrile and loaded onto a homemade reverse-phase microcolumn (Oligo™ R3 Bulk Media for Reversed-Phase Chromatography, 0.5 mm diameter, 25 mm long, 30 µm particle size, Applied Biosystems by Life Technologies, washed with a 0.1% (*v*/*v*) trifluoroacetic acid and separated using step gradient elution as follows: 0.1% (*v*/*v*) trifluoroacetic acid in 10%, 20%, 30%, 40% or 80% (*v*/*v*) aqueous acetonitrile at 20 °C. The separated fractions were directly spotted onto a MALDI grain-steel target (Bruker Daltonics) and after drying were over-laid with alpha-cyano-4-hydroxycinnamic acid matrix (Bruker Daltonics, 5 mg/mL dissolved in 50% (*v*/*v*) aqueous acetonitrile containing 0.1% (*v*/*v*) trifluoroacetic acid). The high resolution MS or MS/MS analyses were acquired on an APEX Qe 9.4T FTICR or Ultraflex III TOF/TOF spectrometer (Bruker Daltonics), respectively. The MS/MS spectra of the cross-linked peptides were interpreted manually.

### 4.7. Molecular Dynamic Calculations

The computational details for all molecular dynamics calculations were published previously [7]. Briefly, the molecular dynamics package Xplor-NIH 2.29 [24,25] and docking, minimization, and rigid body/torsion dynamics protocols were performed in a similar manner as described previously [26]. As the starting structure for the docking calculations, the X-ray structure of 14-3-3ζ (PDB code 3RDH) was used [13]. The calculations are based on 119 distance restraints derived from PDB file plus 4 additional distance restraints (Lys9–Glu81, *N*-terminus–Glu81, Asp21–Lys85, and Glu5–Lys74; so-called “R-6 summed”) from chemical cross-linking [7]. These restrains were allowed to explore the interval of 0 Å to the distance values found in the actual X-ray structure of the protein plus 3 Å. In total, 500 structures were calculated and all structures without distance violations were refined in a layer of explicit water molecules with activated electrostatic potential. The top-ten calculated structures with the lowest potential energy were analyzed with the program MOLMOL 2K.2 [27] and average distances were determined.

## 5. Conclusions

Two recombinant photo-labile 14-3-3ζ protein nanoprobes, WT and S58D, were successfully prepared, and partial incorporation of a photo-labile analog of methionine into both proteins was demonstrated by MS analysis. The covalent link of one molecule of WT photo-14-3-3ζ through its photo-reactive diazirine to another molecule was demonstrated by SDS-PAGE. However, no covalent multimer was detected for the photo-14-3-3ζ S58D, corresponding to its monomeric character. This agrees with the native behaviour of both proteins under native electrophoresis (monomeric photo-14-3-3ζ S58D and dimeric photo-14-3-3ζ WT). The combination of the photo-initiated cross-linking technique with mass spectrometry successfully mapped the regions involved in the homodimerization process of human 14-3-3ζ regulatory protein and revealed the residues in close proximity to each other that participate in the mutual interaction (Gln8–Met78). Moreover, the existence of the detected covalent photo-initiated cross-links cross-validates our previous data using molecular dynamics calculations based on high resolution chemical cross-linking data and H/D exchange mass spectrometry and the 14-3-3ζ X-ray crystal structure (PDB 3DHR). The obtained results verify the successful capability of the emerging photo-initiated cross-linking protein nanoprobe methodology to describe the transient interactions in the protein complexes and to map their interface.
